# Association of Kidney Disease Quality of Life (KDQOL-36) with mortality and hospitalization in older adults receiving hemodialysis

**DOI:** 10.1186/s12882-017-0801-5

**Published:** 2018-01-15

**Authors:** Rasheeda K. Hall, Alison Luciano, Carl Pieper, Cathleen S. Colón-Emeric

**Affiliations:** 1 Durham VA Geriatric Research, Education and Clinical Center, 508 Fulton Street, Durham, NC 27705 USA; 2 Duke University Medical Center, Division of Nephrology, Department of Medicine, Box DUMC 2747, 2424 Erwin Road Suite 605, Durham, NC 27710 USA; 3 Duke University, Department of Biostatistics and Bioinformatics, Box DUMC 2721, 2424 Erwin Road, Suite 1102, Durham, NC 27710 USA; 4 Duke University Medical Center, Division of Geriatrics, Department of Medicine, Box DUMC 3003, Durham, NC 27710 USA

**Keywords:** Geriatric nephrology, Health-related quality of life, Competing risks, SF-12, Prognostication

## Abstract

**Background:**

For older adults receiving dialysis, health-related quality of life is not often considered in prognostication of death or future hospitalizations. To determine if routine health-related quality of life measures may be useful for prognostication, the objective of this study is to determine the extent of association of Kidney Disease Quality of Life (KDQOL-36) subscales with adverse outcomes in older adults receiving dialysis.

**Methods:**

This is a longitudinal study of 3500 adults aged ≥75 years receiving dialysis in the United States in 2012 and 2013. We used Cox and Fine and Gray models to evaluate the association of KDQOL-36 subscales with risk of death and hospitalization. We adjusted for sociodemographic variables, hemodialysis access type, laboratory values, and Charlson index.

**Results:**

Three thousand one hundred thirty-two hemodialysis patients completed the KDQOL-36. From KDQOL-36 completion date in 2012, 880 (28.1%) died and 2023 (64.6%) had at least one hospitalization over a median follow-up of 512 and 203 days, respectively. Cohort members with a SF-12 physical component summary (PCS) in the lowest quintile had an increased adjusted risk of death [hazard ratio (HR), 1.55, 95% confidence interval (CI) 1.19–2.03] and hospitalization (HR, 1.29, 95% CI 1.09–1.54) compared with those with scores in the highest quintile. Cohort members with a SF-12 mental component summary in the lowest quintile had an increased risk of hospitalization (HR, 1.39, 95% CI 1.17–1.65) compared with those in the highest quintile. In adjusted analyses, there was no association between the symptoms of kidney disease, effects of kidney disease, and burden of kidney disease subscales with time to death or first hospitalization. Competing risk models showed similar HRs.

**Conclusions:**

Among the KDQOL-36 subscales, the SF-12 PCS demonstrates the strongest association with both death and future hospitalizations in older adults receiving hemodialysis Further research is needed to assess the value this subscale may add to prognostication.

**Electronic supplementary material:**

The online version of this article (10.1186/s12882-017-0801-5) contains supplementary material, which is available to authorized users.

## Background

Health-related quality of life is increasingly recognized as an important patient-centered outcome for older adults with kidney disease, many of whom have limited life-expectancy and a significant symptom burden [1]. Because health-related quality of life instruments assess self-rated health through items related to physical health, mental health, symptoms and limitations, another potential use for health-related quality of life assessment may be prognostication of adverse outcomes in these patients. Such prognostic information would help clinicians identify a subset of patients at increased risk for death and hospitalization who may benefit from interventions both to reduce the risk of these outcomes and to prepare for treatment decisions that may lie ahead [2, 3]. Several studies in general population cohorts of older adults, older adults with specific chronic conditions, and cohorts of kidney disease in adults of all ages demonstrate the relationship between health-related quality of life and survival [4–18]. To our knowledge, prior studies have not examined the association of health-related quality of life with mortality or hospitalizations in cohorts limited to prevalent older adults receiving maintenance dialysis. While many of the available prognostic tools in older adults with kidney disease include markers suggestive of functional disability (e.g., mobility disability, long-term care, assistance with activities of daily living), none, to our knowledge, have incorporated health-related quality of life [19–23]. Because functional status often determines health-related quality of life and is a stronger predictor of adverse outcomes than disease-specific measures in older adults in the general population [24], we hypothesized that health-related quality of life from the commonly administered Kidney Disease Quality of Life-36 instrument (KDQOL-36) may be useful for prognostication. Towards testing this hypothesis, we first need to determine the extent of association of KDQOL-36 subscale scores with adverse outcomes in older adults. Thus, we conducted a cohort study to determine the strength of the association between subscales on the KDQOL-36 and mortality and hospitalization in a cohort of older adults receiving maintenance dialysis.

## Methods

### Study design and data source

We performed a cohort study using data assembled from a large dialysis organization’s (LDO) clinical records during 2012 and 2013. Derived from the LDO’s census of patients within the United States, this cohort included a nationally representative random sample of 3500 dialysis patients age 75 years and older as of January 1, 2012 who had KDQOL-36 responses in 2012 and were followed until clinical event (death or first hospitalization), or end of the study period, December 31, 2013. For each cohort member, we received non-identifiable patient-level clinical data including dates of clinical events, comorbidities, and laboratory data from January 1, 2012 to December 31, 2013. We selected the cohort size and follow-up period to accommodate financial resource constraints. This study was approved by the Duke University Institutional Review Board.

### Study population

As part of routine clinical care endorsed by the United States’ Centers for Medicare and Medicaid Services (CMS), dialysis staff administered the KDQOL-36 to patients in this cohort at least once a year. Dialysis unit social workers either supplied a paper copy of the KDQOL-36 for self-administration or they helped patients complete it during dialysis if they were unable to self-administer. Of the 3500 patients included in this cohort, we excluded one patient from analyses because multiple KDQOL-36 assessments with inconsistent responses were documented in a single day. An additional 232 (6.6%) patients were excluded because they were missing information necessary for calculation of KDQOL-36 subscale scores (e.g., at least one of the SF-12 items or all items within an individual kidney disease-specific subscale on the KDQOL-36). Because patients receiving peritoneal dialysis (PD) often differ from hemodialysis patients, we excluded an additional 135 cohort members who were receiving PD, resulting in final analytic sample size of 3132. We show characteristics of patients who were included and excluded from analyses in Additional file 1: Table S1.

### Exposure variables

The KDQOL-36 is a 36-item health-related quality of life instrument adapted from the original 134-item KDQOL, an instrument principally developed to measure quality of life of dialysis patients [25]. Because there is no currently accepted overall KDQOL-36 score that incorporates all of its subscales [26], and to be consistent with prior studies [6], we calculated scores for the following five subscales of the KDQOL-36 separately: 1) SF-12 physical component summary (PCS), 2) SF-12 mental component summary (MCS), 3) Burden of kidney disease, 4) Symptoms of kidney disease and 5) and Effects of kidney disease. KDQOL-36 subscale scores ranged from 0 to 100, and lower scores indicated worse self-reported quality of life. These subscale scores were categorized into quintiles (first quintile = lowest scores, fifth quintile = highest scores).

### Covariates

We identified the following baseline characteristics present on the date of KDQOL-36 administration in 2012: age, gender, race (e.g., Caucasian, African-American, Hispanic, or other), whether patients were Medicaid eligible (a proxy for socioeconomic status), Charlson comorbidity index [27],, hemodialysis access type (e.g., arteriovenous fistula, arteriovenous graft, or central venous catheter), and length of time on dialysis (defined as time between each patient’s first dialysis treatment and the date of KDQOL-36 administration during the study period). We also obtained relevant laboratory measures [albumin, hemoglobin, dialysis adequacy (Kt/V)] typically collected less than 1 month from the date of KDQOL-36 administration. For example, mean time between serum albumin levels and KDQOL-36 administration was 13.5 (SD = 10.3) days.

### Outcomes

Outcomes of interest were time to death and time to first hospitalization and were measured from date of KDQOL-36 administration through event date. We defined first hospitalization as the date of a hospitalization occurring in 2012. Among 3132 with complete KDQOL-36 data, five deaths and 37 hospitalizations occurred before the documented date of KDQOL-36 administration, so cohort members with these events were not included in survival analyses.

### Statistical analysis

To determine the extent to which KDQOL-36 subscale scores are associated with death and/or hospitalizations in older adults receiving dialysis, we conducted the following three analyses: 1) a Cox proportional hazards regression model to measure the association of all KDQOL-36 subscales with time to death, 2) a Cox proportional hazards regression model to measure the association of all KDQOL-36 subscales with time to first hospitalization, and 3) a competing risk analysis to measure the association between KDQOL-36 subscales and time to first hospitalization after KDQOL-36 administration, with death as the competing event. In these analyses, baseline socio-demographic (age, sex, race/ethnicity, presence of Medicaid insurance – as a proxy for low socioeconomic status) and clinical (albumin, hemoglobin, Kt/V, Charlson comorbidity index, access type, and time since dialysis initiation) variables were identified a priori as covariates based on previous research [28–31]. Cox analyses were repeated, modeling individual subscales in isolation. We used Lowess curve deviation for horizontal of Martingale residuals to assess linearity of continuous covariates. We assessed categorical covariates for violation of the proportional hazards assumptions (i.e., non-random pattern of Schoenfeld residuals plot versus log time). Because serum albumin did not meet the proportional hazards assumption, we performed stratified Cox models to allow the form of the underlying hazard function to vary across levels of albumin.

For the Cox regression models, subjects were censored at the date of the event (death or first hospitalization after KDQOL-36 administration) or at the end of the study period, December 31, 2013. We evaluated goodness of fit using Schoenfeld residuals, Cox-Snell residuals, and differences in fit due to observation deletion. We assessed the joint relevance of including the KDQOL-36 subscales in models for death and hospitalization by comparing fit between models with and without the subscales to obtain a global likelihood ratio (LR) statistic [32]. We estimated concordance (C-statistic) via Harrell’s C for Cox regression models [33]. For the competing risk analysis, KDQOL-36 assessment was treated as study start date, first hospitalization post assessment as event of interest, and death as competing event. We used the Fine and Gray method to estimate unadjusted and multi-adjusted sub-distribution relative hazards of hospitalization [34, 35]. Surviving participants with no hospitalizations during the study period were right censored. All analyses were performed using Stata version SE 15.

## Results

### Cohort characteristics

Of the 3132 patients in the analytic cohort, at baseline, the average age was 80.5 (4.4) years, 50.1% (*n =* 1570) were male, 22.9% (*n* = 675) received Medicare and Medicaid insurance (dual eligible), time since dialysis initiation was 5.9 (2.9) years, average Charlson index was 7.4 (1.3), and the majority of these (64.5%, *n =* 2018) had an arteriovenous fistula (Table 1). Table 2 shows the distribution of KDQOL-36 subscale scores. No cohort members achieved the maximum score for SF-12 PCS or MCS.

**Table 1:** Baseline Cohort Characteristics

Variable	Total Sample (*n* = 3132)^a^
Age, year	80.5 (4.4)
Race, (*n* = 3131)	
Caucasian	1590 (50.8%)
African-American	893 (28.5%)
Hispanic	420 (13.4%)
Other	228 (7.3%)
Sex	
Women	1562 (49.9%)
Men	1570 (50.1%)
Insurance Status, (*n* = 2945)	
Enrolled in Medicaid	675 (22.9%)
Not Enrolled in Medicaid	2270 (77.1%)
*Medical History*	
Time on Dialysis (years)	5.9 (2.9)
Kt/V^b^, (*n* = 3116)	1.7 (0.3)
Hemoglobin (g/dL)^b^, (*n* = 3131)	10.8 (1.0)
Albumin (gm/dL)^b^, (*n* = 3131)	3.9 (0.3)
Charlson comorbidity index	7.4 (1.3)
Access Type, (*n* = 3131)	
Catheter	294 (9.4%)
Arteriovenous fistula	2018 (64.5%)
Arteriovenous graft	819 (26.2%)

Data expressed as n (%) or mean ± SD

^a^Values based on total of 3132 patients who completed all KDQOL-36 items in 2012, unless otherwise specified

^b^Laboratory values documented closest to the date of the first KDQOL-36 administration in 2012

**Table 2:** Means and Quintile Ranges for Kidney Disease Quality of Life-36 Subscale Scores^a^

	Mean ± SD	1st Quintile	2nd Quintile	3rd Quintile	4th Quintile	5th Quintile
SF-12 Physical Component Score	34.5 ± 9.9	11.9 to 25.5 (N = 627)	25.5 to 30.4 (N = 626)	30.4 to 36.5 (*N* = 627)	36.5 to 43.8 (*N* = 626)	43.8 to 63.6 (N = 626)
SF-12 Mental Component Score	50.9 ± 10.4	14.6 to 40.9 (N = 627)	40.9 to 49.4 (N = 626)	49.4 to 55.8 (N = 627)	55.8 to 60.6 (N = 626)	60.6 to 72.2 (N = 626)
Symptoms/Problems	78.5 ± 15.6	14.6 to 65.9 (N = 627)	66.7 to 77.1 (*N* = 654)	77.3 to 85.4 (*N* = 689)	86.4 to 91.7 (*N* = 598)	92.5 to 100.0 (*N* = 564)
Effects of Kidney Disease	74.3 ± 21.3	0.0 to 56.3 (*N* = 674)	57.1 to 71.9 (*N* = 637)	75.0 to 84.4 (*N* = 649)	85.0 to 93.8 (*N* = 611)	95.0 to 100.0 (*N* = 561)
Burden of Kidney Disease	52.6 ± 29.4	0.0 to 25.0 (*N* = 814)	31.3 to 43.8 (*N* = 587)	50.0 to 62.5 (*N* = 600)	66.7 to 81.3 (*N* = 585)	87.5 to 100.0 (*N* = 546)

^a^Values from minimum to maximum for each quintile are shown. *N* = 3132

### Association between KDQOL-36 subscales and mortality

**Fig. 1:**
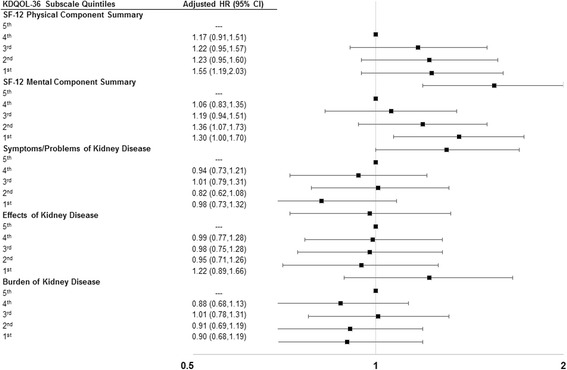
Adjusted Hazard Ratios for Mortality based on Cox Regression model^a^. ^a^Model included five subscales (presented) and the following adjustment factors as of first KDQOL-36 administration after January 1, 2012: age, race, sex, Medicaid status, time on dialysis (years), Kt/V, hemoglobin, charlson comorbidity index score, and access type (catheter, arterialvenous fistula, arterialvenous graft). Model results (hazard ratios and 95% confidence intervals) for covariates presented in Additional file 1: Table S4

Over the study period, 28.1% (*n =* 880) of the cohort members died after date of completion of the KDQOL-36 (median follow-up of 512, range, 1–730) days. In unadjusted analyses, cohort members in the first quintile (relative to the fifth quintile) for all five KDQOL-36 subscales had a higher hazard of death (Additional file 1: Table S2). After adjustment for sociodemographic characteristics, time since dialysis initiation, Charlson index, access type, and laboratory values, these associations were attenuated (Additional file 1: Table S3). With all five KDQOL-36 subscales combined in a model with covariates, only cohort members in the first quintile for the SF-12 PCS maintained a significantly higher hazard of death [hazard ratio (HR), 1.55, 95% confidence interval (CI) 1.19–2.03] when compared with patients in the fifth quintile (Fig. 1). In this model, race, gender, time since dialysis initiation, hemoglobin, and Charlson index also maintained significant associations with time to death (Additional file 1: Table S4). Compared to a regression model that included sociodemographic characteristics, time since dialysis initiation, Charlson index, access type, and laboratory values, overall model fit improved with block addition of the five KDQOL-36 subscales (LR Χ^2^ (df = 20)) = 42.64; *p*-value = 0.002). The C-statistic w/ bootstrapping for this model with all five KDQOL-36 subscales was 0.63 (95% CI 0.61–0.65).

### Associations of KDQOL-36 subscales with first hospitalization

Over the study period, 64.6% (*n* = 2023) of the cohort members were hospitalized at least once after date of completion of the KDQOL-36 (median follow-up of 203 (range, 1–730) days. Among those who were hospitalized, 730 (36.1%) later died during the observation period. However, 92.2% (*n* = 673) died at least 7 days after the hospitalization. The average length of stay was 5.6 (7.6) days. Although nearly 50% of hospitalization diagnoses were unavailable, the most common causes of hospitalization that were documented were shortness of breath (*n* = 154, 7.6%), pneumonia (*n* = 105, 5.2%), and chest pain (*n* = 94, 4.6%). In unadjusted analyses, cohort members in the first quintile (relative to the fifth quintile) for all five KDQOL-36 subscales had a higher hazard of hospitalization (Additional file 1: Table S2). After adjustment for sociodemographic characteristics, time since dialysis initiation, Charlson index, access type, and laboratory values, these associations were attenuated (Additional file 1: Table S5). With all five KDQOL-36 subscales combined in a model with covariates, cohort members in the first quintile (relative to the fifth quintile) for the SF-12 MCS (HR, 1.39, 95% CI 1.17–1.65) and SF-12 PCS (HR, 1.29, 95% CI 1.09–1.54) maintained a higher hazard of hospitalization (Table 3). Compared to a regression model that included sociodemographic characteristics, time since dialysis initiation, Charlson index, access type, and laboratory values, overall model fit improved with block addition of the five KDQOL-36 subscales (LR Χ^2^ (df = 20)) = 62.33; *p*-value <0.001). The C-statistic w/ bootstrapping for this model with all five KDQOL-36 subscales was 0.58 (95% CI, 0.56–0.59). In the competing risk model, these associations were similar [SF-12 MCS, subdistribution hazard ratio (sdHR), 1.38, 95% CI 1.16–1.64; SF-12 PCS, sdHR, 1.31, 95% CI 1.10–1.56] (Table 3).

**Table 3:** Adjusted Analyses for Risk of Hospitalization with and without Competing Mortality

Quintile	Adjusted HR (95% CI)	Adjusted sdHR (95% CI)
Physical		
1st	1.29 (1.09, 1.54)	1.31 (1.10, 1.56)
2nd	1.13 (0.96, 1.33)	1.14 (0.96, 1.35)
3rd	1.07 (0.91, 1.26)	1.07 (0.91, 1.26)
4th	1.12 (0.96, 1.30)	1.13 (0.97, 1.32)
5th	–	–
Mental		
1st	1.39 (1.17, 1.65)	1.38 (1.16, 1.64)
2nd	1.20 (1.02, 1.40)	1.20 (1.02, 1.40)
3rd	1.10 (0.95, 1.28)	1.08 (0.93, 1.26)
4th	1.03 (0.89, 1.20)	1.03 (0.89, 1.19)
5th	–	–
Symptoms		
1st	1.14 (0.94, 1.38)	1.16 (0.96, 1.41)
2nd	0.94 (0.78, 1.12)	0.94 (0.79, 1.13)
3rd	1.02 (0.86, 1.20)	1.02 (0.87, 1.21)
4th	0.92 (0.78, 1.07)	0.91 (0.78, 1.06)
5th	–	–
Effects		
1st	0.82 (0.67, 1.01)	0.80 (0.65, 0.99)
2nd	0.90 (0.75, 1.08)	0.87 (0.72, 1.05)
3rd	0.94 (0.79, 1.11)	0.94 (0.79, 1.12)
4th	0.88 (0.75, 1.04)	0.88 (0.75, 1.04)
5th	–	–
Burden		
1st	1.00 (0.84, 1.20)	1.01 (0.84, 1.21)
2nd	1.08 (0.91, 1.28)	1.07 (0.90, 1.27)
3rd	0.92 (0.78, 1.09)	0.92 (0.78, 1.09)
4th	0.99 (0.85, 1.16)	0.98 (0.84, 1.15)
5th	–	–
Age, years	1 (0.99, 1.01)	1 (0.99, 1.01)
Race		
Caucasian	–	–
African-American	0.86 (0.77, 0.96)	0.86 (0.77, 0.96)
Hispanic	0.85 (0.73, 0.98)	0.85 (0.73, 0.98)
Other	0.62 (0.51, 0.76)	0.62 (0.50, 0.76)
Sex		
Female	–	–
Male	0.92 (0.84, 1.02)	0.91 (0.83, 1.01)
Enrolled in Medicaid		
No	–	–
Yes	1.06 (0.94, 1.19)	1.07 (0.95, 1.20)
Time on Dialysis (years)	1.01 (1.00, 1.03)	1.01 (1.00, 1.03)
Kt/V	0.92 (0.78, 1.08)	0.91 (0.77, 1.07)
Hemoglobin (g/dL)	0.91 (0.86, 0.96)	0.90 (0.85, 0.95)
Charlson comorbidity index	1.05 (1.02, 1.09)	1.06 (1.02, 1.09)
Access Type		
Catheter	–	–
Arterialvenous fistula	0.88 (0.75, 1.03)	0.86 (0.73, 1.02)
Arterialvenous graft	0.94 (0.79, 1.12)	0.93 (0.78, 1.10)

Data expressed as hazard ratio (HR) for Cox Proportional Hazards Model [or subdistribution hazard ratio (sdHR) for competing risk model] and 95% confidence interval (CI). Significant hazard ratios (*p* < .05) indicated in bold

Analyses conducted on complete cases, *n* = 2895, and were adjusted for all KDQOL-36 subscales and covariates shown in table

## Discussion

A growing number of patients treated with maintenance dialysis are older, frail and have functional impairment [29, 36]. Among populations of older adults in the general population, the presence of functional impairment is often a more powerful predictor of adverse outcomes than traditional disease-based measures [24]. Better prognostic tools that incorporate information on functional status could be helpful in supporting discussions about prognosis, treatment preferences and goals of care for many of these vulnerable patients. As a first step, the current study estimated the association between a patient-reported outcome measure that is efficiently and reliably collected as part of routine clinical care, the KDQOL-36 subscales, and two important and easily measured endpoints: mortality and hospitalization. Among KDQOL-36 subscales only SF-12 PCS demonstrates significant associations with both mortality and hospitalization risk. If combined with other prognostic tools, this subscale may yield a modest improvement in prediction of remaining lifetime and future hospitalizations in older adults receiving dialysis. Although many older adults receiving maintenance dialysis have limited life expectancy and significant comorbidity burden, as reflected in the generally low health-related quality of life observed in this population, their SF-12 PCS responses were independently associated with mortality. To date, there are no observational studies or clinical practice guidelines to guide how low self-reported physical health (or SF-12 PCS) should impact care for dialysis patients. However, our study demonstrates that this patient-reported information could add a distinct construct to existing prediction tools that rely on clinical gestalt and/or comorbidities, lab values, and crude measures of functional status (e.g., impaired mobility or dependency in activities of daily livings) [3]. Our results in patients aged ≥75 years extends that of other studies demonstrating that the SF-12 PCS (or its larger form, SF-36 PCS) is associated with survival in younger patients receiving dialysis, patients with CKD, community dwelling older adults, and older adults with other chronic conditions [6–10, 13, 37]. Similar to a large Dialysis Outcomes and Practice Patterns Study that evaluated the association of KDQOL-SF (instead of KDQOL-36) subscales with mortality in a younger cohort (mean age, 60.5 ± 15.2) [8], we also identified weaker or non-existent associations of the SF-12 MCS and the kidney disease-specific subscales with mortality, suggesting these subscales, compared to SF-12 PCS, may be less likely to improve efforts to predict the probability of mortality in older adults receiving dialysis [38].

Consistent with prior evidence in a wider age range of the dialysis population [8], our analyses reveal that hospitalizations occur more quickly among cohort members who report low self-rated health on the SF-12 [39]. This association may be explained by frailty as it often contributes to limited physical function, and increases vulnerability to acute stressors that warrant hospitalization [36, 40]. Nearly two-thirds of the cohort experienced a hospitalization in 2012 which underscores the importance of developing interventions that add additional layers of support for high-risk patients at home and other non-acute settings. Our findings suggest that adding the SF-12 to risk factors identified in prior studies [41, 42], may offer a modest improvement in identification of high risk patients who may benefit from additional intervention and/or oversight to proactively address concerns early when they first arise and prevent hospitalizations. Because these patients tend to have multiple comorbidities, additional research on the role of distinct comorbidities (e.g., heart failure or cancer) on KDQOL-36 subscales may also enhance our ability to identify patients at risk of frequent hospitalizations for management of those comorbidities.

Although we identified association of SF-12 with death and/or hospitalization, the SF-12, as well as the other KDQOL-36 subscales, appear to only yield a modest improvement in prognostication of those outcomes in older dialysis patients. While additional studies are needed to confirm our findings for development of prognostic tools (e.g., external validation), our findings also suggest that aspects of health related quality of life that are most associated with adverse outcomes in older adults are not adequately measured by the KDQOL-36. The KDQOL-36 does not capture aspects of quality of life that are valued by older adults, such as social relationships, financial circumstances, independence, and positive outlook [43]. These aspects are measured in the Older People’s Quality of Life Questionnaire (OPQOL) [43], and may enhance both model fit and prognostic accuracy. Additional research into the relationship of the OPQOL or other older adult-specific instruments with death and hospitalization in older dialysis patients is needed.

Our study is the first to evaluate the relationship of KDQOL-36 subscales with adverse outcomes in a cohort limited to older adults receiving dialysis. However, there are limitations to consider when interpreting our results. First, we derived our cohort from a single LDO that provides for-profit dialysis services to more than one-third of the United States’ dialysis population, and the average time on dialysis for cohort members was nearly 6 years. Thus, our results may not be generalizable to all dialysis patients aged ≥75 years worldwide, particularly those who are new to dialysis, as well as, those receiving dialysis in the United States’ non-profit dialysis organizations or federal programs. Still, we find the cohort characteristics to be similar to those for the overall population of older adults receiving maintenance dialysis registered in the United States Renal Data System [44]. Second, we selected a cohort who completed the KDQOL-36; therefore, these findings have limited generalizability to patients who are unable to complete the KDQOL-36, particularly older adults with dementia. For this subset of patients, a separate analysis using a HRQOL tool specific for people with dementia is more ideal because dementia contributes its own significant association with death and dialysis withdrawal rates [45, 46]. Third, not all unmeasured confounders or prognostic variables identified in previous research were available in the dataset, including new medical diagnoses, medications, blood pressure, weight gain, lean body mass, depression, social support, level of care (e.g., self-care or skilled nursing) and prior hospitalizations [47–49]. Fourth, we had insufficient data to report the most common reasons for hospitalization or death. Such granular data would be helpful in subsequent studies to elucidate if low health-related quality of life is associated with the onset of specific health events (e.g., cardiovascular event, including related death, or access-related infection). Finally, our analyses do not capture serial KDQOL-36 data so we did not test the association of worsening quality of life or functional status on mortality and hospitalizations. However, recent evidence suggests that a patient’s most recent measure of quality of life is more predictive of mortality than the change in quality of life over time [37]. Finally, we acknowledge measurement error from the KDQOL-36 may bias our findings.

## Conclusion

We report that among KDQOL-36 subscales, SF-12 PCS, has the potential to enhance prognostication of survival and future hospitalizations in older adults receiving dialysis. If validated in other studies, this finding may support routine quality of life assessments and integration of health-related quality of life and clinical data into prediction tools that ultimately enhance risk stratification and shared decision-making for older adult receiving maintenance dialysis.

## Additional file

Additional file 1: Table S1. Baseline characteristics of cohort members included and excluded from analytic sample. **Table S2.** Unadjusted Hazard Ratios for Death and Hospitalization and Subdistribution Hazard Ratios for Hospitalization. **Table S3.** Adjusted regression models predicting death by quantile KDQOL-36 subscale score. **Table S4.** Effect Estimates for Covariates of Cox Model for Risk of Death. **Table S5.** Adjusted regression models predicting hospitalization by quantile KDQOL-36 subscale score. (DOCX 25 kb)

## Abbreviations

AVF: Arteriovenous fistula
AVG: Arteriovenous graft
CMS: Centers for Medicare and Medicaid Services
KDQOL-36: Kidney Disease Quality of Life – 36 item instrument
LDO: Large dialysis organization
MCS: Mental component score
PCS: Physical component score
PD: Peritoneal dialysis
SD: Standard deviation
SF-12: Short Form – 12 item instrument
U. S.: United States
