# Supplementary material for: Association of Kidney Disease Quality of Life (KDQOL-36) with mortality and hospitalization in older adults receiving hemodialysis
Source: BMC Nephrol. 2018 Jan 15;19:11. doi: 10.1186/s12882-017-0801-5 (PMC5769495; doi:10.1186/s12882-017-0801-5)
Supplement: Additional file 1: Table S1. — Baseline characteristics of cohort members included and excluded from analytic sample. Table S2. Unadjusted Hazard Ratios for Death and Hospitalization and Subdistribution Hazard Ratios for Hospitalization. Table S3. Adjusted regression models predicting death by quantile KDQOL-36 subscale score. Table S4. Effect Estimates for Covariates of Cox Model for Risk of Death. Table S5. Adjusted regression models predicting hospitalization by quantile KDQOL-36 subscale score. (DOCX 25 kb) [file 12882_2017_801_MOESM1_ESM.docx]

**Table S1** Baseline Characteristics of cohort members included and excluded from analytic sample

| Variable | Total Sample (N = 3,500) | Included (N = 3,132) | Excluded^a^ (N = 368) |
| --- | --- | --- | --- |
| Age, year | 80.5±4.4 | 80.5±4.4 | 80.5±4.3 |
| Race, (*n* = 3,499) |  |  |  |
| Caucasian | 1,786 (51.0%) | 1590 (50.8%) | 196 (53.3%) |
| African-American | 988 (28.2%) | 893 (28.5%) | 95 (25.8%) |
| Hispanic | 466 (13.3%) | 420 (13.4%) | 46 (12.5%) |
| Other | 259 (7.4%) | 228 (7.3%) | 31 (8.4%) |
| Men | 1,770 (50.6%) | 1570 (50.1%) | 200 (54.3%) |
| Insurance Status (*n* = 3,289) |  |  |  |
| Enrolled in Medicaid | 738 (22.4%) | 675 (22.9%) | 63 (18.3%) |
| Not Enrolled in Medicaid | 2,551 (77.6%) | 2270 (77.1%) | 281 (81.7%) |
| *Medical History* |  |  |  |
| Time on Dialysis (years), (*n* = 3,499) | 5.9±2.9 | 5.9±2.9 | 5.5±2.4 |
| Kt/V^b^, (*n* = 3,475) | 1.7±0.3 | 1.7±0.3 | 1.9±0.5 |
| Hemoglobin (g/dL) ^b^, (*n* = 3,497) | 10.8±1.0 | 10.8±1.0 | 10.9±1.1 |
| Albumin (gm/dL) ^b^, (*n* = 3,497) | 3.9±0.4 | 3.9±0.4 | 3.7±0.4 |
| Charlson comorbidity index, (*n* = 3,500) | 7.4±1.3 | 7.4±1.3 | 7.3±1.3 |
| Access Type (*n* = 3,357) |  |  |  |
| Catheter | 318 (9.5%) | 294 (9.4%) | 24 (10.6%) |
| Arteriovenous fistula | 2,160 (64.3%) | 2018 (64.5%) | 142 (62.8%) |
| Arteriovenous graft | 879 (26.2%) | 819 (26.2%) | 60 (26.5%) |

Data expressed as n (%) or mean ± SD based on total of 3,500 patients, unless otherwise specified.

^a^Excluded cohort members either did not have sufficient information for KDQOL-36 subscale scores or were receiving peritoneal dialysis at the time of KDQOL-36 administration.

^b^Laboratory values were values documented closest to the date of the first KDQOL-36 administration in 2012.

**Table S2** Unadjusted Hazard Ratios for Death and Hospitalization and Subdistribution Hazard Ratios for Hospitalization

| Quintile | A.  HR for Death  (95% CI)^a^ | B.  HR for First Hospitalization  (95% CI)^b^ | C.  sdHR for First Hospitalization  (95% CI)^c^ |
| --- | --- | --- | --- |
| Physical Component | | | |
| 1st | 1.95 (1.57,2.42) | 1.50 (1.31,1.73) | 1.47 (1.28,1.68) |
| 2nd | 1.59 (1.27,1.98) | 1.26 (1.10,1.46) | 1.24 (1.08,1.43) |
| 3rd | 1.48 (1.18,1.85) | 1.15 (1.00,1.33) | 1.14 (0.99,1.32) |
| 4th | 1.31 (1.04,1.66) | 1.18 (1.02,1.36) | 1.17 (1.02,1.35) |
| 5th | --------- | --------- | --------- |
| Mental Component |  |  |  |
| 1st | 1.39 (1.13,1.72) | 1.41 (1.23,1.62) | 1.39 (1.21,1.59) |
| 2nd | 1.23 (0.99,1.52) | 1.13 (0.99,1.30) | 1.13 (0.99,1.30) |
| 3rd | 1.13 (0.91,1.40) | 1.06 (0.92,1.22) | 1.05 (0.91,1.21) |
| 4th | 1.00 (0.80,1.25) | 0.99 (0.86,1.14) | 0.99 (0.86,1.14) |
| 5th | --------- | --------- | --------- |
| Symptoms/Problems |  |  |  |
| 1st | 1.47 (1.19,1.83) | 1.39 (1.21,1.60) | 1.38 (1.20,1.58) |
| 2nd | 1.16 (0.93,1.45) | 1.11 (0.96,1.28) | 1.10 (0.95,1.27) |
| 3rd | 1.22 (0.98,1.52) | 1.11 (0.96,1.27) | 1.10 (0.96,1.27) |
| 4th | 1.06 (0.84,1.33) | 0.96 (0.83,1.11) | 0.94 (0.82,1.09) |
| 5th | --------- | --------- | --------- |
| Effects of Kidney Disease |  |  |  |
| 1st | 1.64 (1.33,2.03) | 1.18 (1.02,1.35) | 1.16 (1.01,1.34) |
| 2nd | 1.21 (0.96,1.51) | 1.04 (0.90,1.19) | 1.03 (0.89,1.19) |
| 3rd | 1.24 (0.99,1.55) | 1.08 (0.94,1.25) | 1.08 (0.94,1.25) |
| 4th | 1.13 (0.90,1.43) | 0.92 (0.80,1.07) | 0.93 (0.80,1.07) |
| 5th | --------- | --------- | --------- |
| Burden of Kidney Disease |  |  |  |
| 1st | 1.38 (1.12,1.70) | 1.21 (1.06,1.39) | 1.20 (1.05,1.37) |
| 2nd | 1.29 (1.03,1.61) | 1.21 (1.05,1.40) | 1.19 (1.03,1.38) |
| 3rd | 1.25 (1.00,1.57) | 0.99 (0.85,1.14) | 0.98 (0.85,1.13) |
| 4th | 1.02 (0.80,1.29) | 1.04 (0.90,1.21) | 1.04 (0.89,1.20) |
| 5th | --------- | --------- | --------- |

Data expressed as hazard ratio (HR) for Cox Proportional Hazards Model [or subdistribution hazard ratio (sdHR) for competing risk model] and 95% confidence interval (CI). Significant hazard ratios (p<.05) indicated in bold.

^a^Unadjusted Cox regression models for death; *n* = 3,127

^b^Unadjusted Cox regression models for first hospitalization; *n* = 3,095

^c^Unadjusted Fine and Gray regression models for first hospitalization; *n* = 3,095

**Table S3** Adjusted Regression Models for **Death** by KDQOL-36 Subscale Quantile

|  | Physical Component  Summary | Mental Component  Summary | Symptoms/Problems of Kidney Disease | Effects of Kidney Disease | Burden of Kidney Disease |
| --- | --- | --- | --- | --- | --- |
|  | **Adjusted HR (95% CI)** | | | | |
| KDQOL-36 Subscale Quantile | | | | | |
| 1st | 1.72 (1.37,2.16) | 1.48 (1.19,1.84) | 1.47 (1.18,1.84) | 1.53 (1.22,1.91) | 1.29 (1.04,1.61) |
| 2nd | 1.35 (1.07,1.70) | 1.37 (1.10,1.71) | 1.07 (0.85,1.36) | 1.11 (0.88,1.41) | 1.15 (0.91,1.46) |
| 3rd | 1.3 (1.03,1.64) | 1.21 (0.97,1.51) | 1.2 (0.96,1.51) | 1.09 (0.86,1.39) | 1.16 (0.92,1.47) |
| 4th | 1.21 (0.95,1.54) | 1.04 (0.82,1.31) | 1.03 (0.81,1.31) | 1.04 (0.82,1.33) | 0.94 (0.73,1.20) |
| 5th | --- | --- | --- | --- | --- |
| *Model Fit Statistics* | | | | | |
| Harrell's C | 0.63 | 0.63 | 0.63 | 0.62 | 0.62 |

N = 2,923; Models included the following adjustment factors as of first KDQOL-36 administration after January 1, 2012: age, race, sex, Medicaid status, time on dialysis (years), Kt/V, hemoglobin, charlson comorbidity index score, and access type (catheter, arteriovenous fistula, arteriovenous graft).

**Table S4** Effect Estimates for Covariates of Cox Model for Risk of Death

|  | **Adjusted HR (95% CI)** | |
| --- | --- | --- |
| Age, years | 1.03 (1.02,1.05) |  |
| Race |  |  |
| Caucasian | --- |  |
| African-American | 0.58 (0.49,0.70) |  |
| Hispanic | 0.69 (0.55,0.87) |  |
| Other | 0.57 (0.42,0.79) |  |
| Sex |  |  |
| Female | --- |  |
| Male | 1.20 (1.03,1.40) |  |
| Enrolled in Medicaid |  | |
| No | --- |  |
| Yes | 1.08 (0.90,1.30) |  |
| Time on Dialysis (years) | 1.03 (1.01,1.06) |  |
| Kt/V | 0.88 (0.69,1.13) |  |
| Hemoglobin (g/dL) | 0.89 (0.82,0.96) |  |
| Charlson comorbidity index | 1.12 (1.06,1.18) |  |
| Access Type |  | |
| Catheter | --- |  |
| Arteriovenous fistula | 0.81 (0.64,1.02) |  |
| Arteriovenous graft | 1.01 (0.78,1.29) |  |

N=2,923; Data expressed as hazard ratio (HR) for Cox Proportional Hazards Model and 95% confidence interval (CI).

**Table S5** Adjusted Regression Models for **Hospitalization** by KDQOL-36 Subscale Quantile

N = 2,895; Models included the following adjustment factors as of first KDQOL-36 administration after January 1, 2012: age, race, sex, Medicaid status, time on dialysis (years), Kt/V, hemoglobin, charlson comorbidity index score, and access type (catheter, arteriovenous fistula, arteriovenous graft).

|  | Physical Component  Summary | Mental Component  Summary | Symptoms/Problems of Kidney Disease | Effects of Kidney Disease | Burden of Kidney Disease |
| --- | --- | --- | --- | --- | --- |
|  | **Adjusted HR (95% CI)** | | | | |
| KDQOL-36 Subscale Quantile | | | | | |
| 1st | 1.38 (1.20,1.60) | 1.45 (1.26,1.67) | 1.36 (1.18,1.58) | 1.16 (1.01,1.35) | 1.24 (1.08,1.43) |
| 2nd | 1.22 (1.06,1.42) | 1.19 (1.03,1.38) | 1.04 (0.90,1.21) | 1.08 (0.93,1.25) | 1.22 (1.05,1.42) |
| 3rd | 1.11 (0.95,1.28) | 1.09 (0.95,1.27) | 1.08 (0.94,1.25) | 1.06 (0.92,1.23) | 1.00 (0.86,1.16) |
| 4th | 1.15 (1.00,1.34) | 1.01 (0.88,1.18) | 0.94 (0.81,1.10) | 0.92 (0.79,1.07) | 1.03 (0.88,1.20) |
| 5th | --- | --- | --- | --- | --- |
| *Model Fit Statistics* | | | | | |
| Harrell's C | 0.57 | 0.57 | 0.57 | 0.57 | 0.56 |
